# MANAGEMENT OF SYNCHRONIC LARGE LIVER METASTASIS IN A NON-OCCLUSIVE COLON TUMOR

**DOI:** 10.1590/0102-6720202400064e1858

**Published:** 2025-01-20

**Authors:** Eduardo José Brommelstroet Ramos, Hugo Pinto Marques, Martin Palavecino, Timothy Pawlik, Rene Adam, Olivier Soubrane, Paulo Herman, Ricardo Lemos Cotta-Pereira

**Affiliations:** 1 Universidade Federal do Paraná – Curitiba (PR), Brasil; 2 Centro Hospitalar Universitário de Lisboa Central, Curry Cabral Hospital, Hepato-Biliary-Pancreatic and Transplantation Centre – Lisbon, Portugal; 3 Hospital Italiano de Buenos Aires, General Surgery Unit – Buenos Aires, Argentina; 4 Ohio State University, Wexner Medical Center, Department of Surgery – Columbus (OH), USA; 5 University Paris-Saclay, AP-HP Paul Brousse Hospital, Hepato Biliary Surgery, Cancer and Transplantation Unit – Villejuif, France; 6 Universite Paris Descartes, Institute Mutualiste Montsouris, Oncologic and Metabolic Surgery, Department of Digestive – Paris, France; 7 Universidade de São Paulo, Faculty of Medicine, Department of Gastroenterology, – São Paulo (SP), Brazil; 8 D’Or Institute for Research and Education, Digestive Surgery Residency Program – Rio de Janeiro (RJ), Brazil.

**Keywords:** Neoplasm metastasis, Liver, Colorectal neoplasms, Chemotherapy, Hepatectomy, Metástase neoplásica, Fígado, Neoplasias colorretais, Quimioterapia, Hepatectomia

## Abstract

In patients with synchronic liver colorectal metastasis, resection of the primary tumor and liver metastases is the only potentially curative strategy. In such cases, there is no consensus on whether resection of the primary tumor and metastases should be performed simultaneously or whether a staged approach should be performed (resection of the primary tumor and after, hepatectomy, or hepatectomy first). Patients with no bowel occlusion and with extensive liver disease are advised neoadjuvant oncological therapy. Similarly, various strategies such as portal vein embolization, liver deprivation, two-staged hepatectomy, and associating liver partition and portal vein ligation are available for patients who do not have a sufficient future liver remnant (generally 30-40% of the total). Therefore, a multidisciplinary approach is required for the treatment of these patients.

## RESUMO

Pacientes com metástase colorretal hepática sincrônica, a ressecção do tumor primário e das metástases hepáticas é a única estratégia potencialmente curativa. Nesses casos, não há consenso sobre se a ressecção do tumor primário e das metástases deve ser realizada simultaneamente ou se uma abordagem em estágios deve ser realizada (ressecção do tumor primário e depois, hepatectomia ou hepatectomia primeiro). Pacientes que não têm oclusão intestinal e têm uma doença hepática extensa são encaminhados para terapia oncológica neoadjuvante. Quando o paciente não tem remanescente hepático futuro suficiente (geralmente 30–40% do total), muitas estratégias podem ser usadas: embolização da veia porta, privação hepática, hepatectomia em dois estágios e ALPPS (partição do fígado e a ligadura da veia porta). Portanto, uma abordagem multidisciplinar é necessária no tratamento desses pacientes.

## INTRODUCTION

Colorectal cancer is the second most common solid organ cancer. Traditional treatment includes surgery and chemotherapy. In patients with synchronic liver metastasis, resection of the primary tumor and liver metastases is the only potentially curative strategy. In such cases, there is no consensus on whether resection of the primary tumor and metastases should be performed simultaneously or whether a staged approach should be performed (resection of the primary tumor and after, hepatectomy, or the "liver-first" approach)^4,5^.

### Surgical technique

A 72-year-old woman presented with weakness, loss of appetite, and mild abdominal pain. She denied nausea, vomiting, and bowel complaints. There was a past medical history of hypertension, hypothyroidism, and dyslipidemia. Current medications were levothyroxine, simvastatin, and valsartan. The patient also reported a past surgical history of tubal ligation and abdominoplasty. Abdominal ultrasound on 01/16/17 showed a hypoechoic mass of 95 cm × 89 cm × 85 cm in segments IV, V, VI, and VIII. The patient was referred to a tertiary hospital with a specialized team.

**Figure 1:**
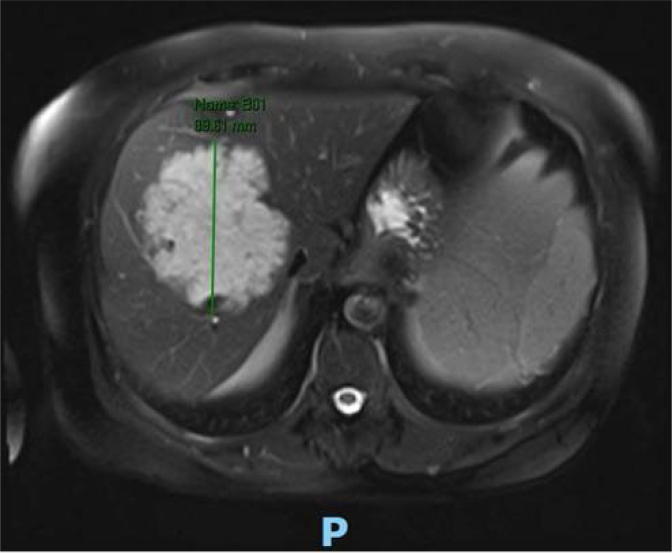
Lesion centered in segment VIII, with 95 × 89 × 85 cm.

**Figure 2:**
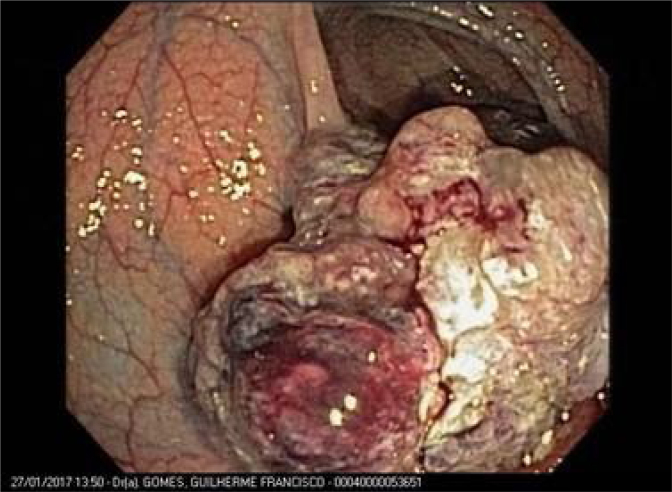
A vegetative non-occlusive lesion in the colonic splenic angle.

Abdominal magnetic resonance imaging (MRI) was performed with an intravenous hepatocyte-specific paramagnetic contrast which showed a liver with normal topography and contours, presenting a large expansive lesion, with lobulated contours, hyperintense on T2, with apparently continuous enhancement in the periphery, without retention in the hepatobiliary phase, predominantly involving segment VIII, however extending to segments IV, V, and VII. The lesion measured approximately 9.5 cm × 8.9 cm × 8.5 cm (Figure 1). There was also a lesion with a vegetative appearance, hyperintense on T2, located in the lumen of the colon in the region of the splenic angle, measuring approximately 5.9 cm × 4.5 cm. Colonoscopy revealed an 8-cm non-occlusive lesion in the splenic angle (Figure 2). Pathology showed a well-differentiated tubular papillary adenocarcinoma, wild-type KRAS. Carcinoembryonic antigen (CEA) was 69 ng/mL.

The patient was discussed in a multidisciplinary setting, and it was decided to start chemotherapy with FOLFOX (leucovorin calcium, 5-fluororacil, and oxaliplatin). The clinical evaluation classified the patient as Eastern Cooperative Oncology Group 1 (ECOG 1) to tolerate chemotherapy. Then, from January 2017 to March 2017, the patient received four cycles of mFOLFOX6 (association with cetuximab and simvastatin).

A new MRI in March 2017 did not show a significant difference in tumor size. It showed an expansive liver lesion of 9.3 cm × 8.7 cm × 8.5 cm, without changes compared to the previous examination, no signs of retroperitoneal adenopathy, and a small decrease in the colonic lesion, currently measuring about 4.5 cm × 3.5 cm. The CEA level decreased to 13 ng/mL.

**Figure 3:**
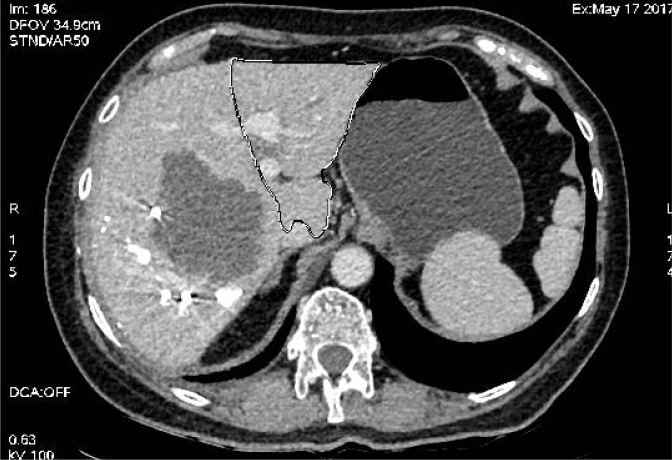
Computed tomography scan post portal vein embolization, with remnant liver volume of 22%.

**Figure 4:**
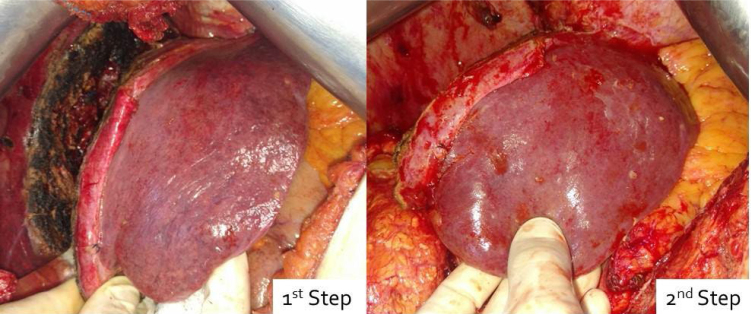
Left lateral segment at the first and second steps of the associate liver partition and portal vein ligation for staged hepatectomy procedure.

The patient was again discussed in the multidisciplinary setting and was decided for right portal vein embolization, which was performed in April 2017, and it was decided to maintain the same type of chemotherapy. Abdominal computed tomography (CT) performed in May 2017, 5 weeks after portal vein embolization, revealed a small decrease in the lesion size, with 8.5 cm × 8.2 cm, and a remnant liver volume (RLV) of 22% (Figure 3). At this point, the patient received a total of eight cycles of chemotherapy. In this setting, it was decided to associate liver partition and portal vein ligation for staged hepatectomy (ALPPS). The ALPPS steps were performed in June 2012 (Figure 4), and a CT performed 2 weeks later showed an increase in the RLV of up to 33%. Right tri-segmentectomy was performed 17 days after the first surgery. The patient was discharged on postoperative day 14, with no major complications. After recovery from the liver surgery, the patient underwent a partial colectomy in August 2017. Pathology revealed a moderately differentiated adenocarcinoma, invasive and ulcerated, with 30% of mucinous areas. There was a lesion of 5.5 cm, with no lymph node involvement (0/12), and with T3N0 staging. The patient was decided on complete chemotherapy with capecitabine from September 2017 to January 2018 and remains free of diseases as of February 2023.

## DISCUSSION

Due to improvements in surgical techniques and the diversity of available systemic and locoregional treatments, patients with synchronic liver metastasis need to be discussed in a multidisciplinary setting, to decide about neoadjuvant chemotherapy, the number of cycles, and the time required to perform colonic and liver resections^3,5^. First reported in 2012, the ALPPS procedure is one of the options to stimulate rapid and marked FLR hypertrophy and to minimize the risk of postoperative liver failure^1,2,6–8^.

### Learning and Discussion points

- Management of synchronic liver metastasis
- Liver first vs. colon first
- Neoadjuvant or adjuvant chemotherapy in liver metastasis. When, how, and for how long time to do.
- Advantages and results of portal vein embolization vs. liver deprivation.
- Two-stage hepatectomy vs. ALPPS procedure

Patients must be well selected for each strategy, and many factors can influence the decisions: presence of bilobar disease, FLR, presence of colonic bleeding or obstruction, patient condition, and specialized hospital team experience. In general, when more than four liver segments need to be resected, patients are not selected for simultaneous liver and colon resections.

## CONCLUSIONS

Patients who do not have bowel occlusion and who have extensive liver disease are advised neoadjuvant oncological therapy. Similarly, various strategies such as portal vein embolization, liver deprivation, two-staged hepatectomy, and ALPPS can be used when patients do not have a sufficient FLR (generally 30-40% of the total). Therefore, a multidisciplinary approach is required for the treatment of these patients.

## AUTHORS' COMMENT

Rene Adam: "This patient history is interesting, because many, many teams would have proposed a liver biopsy to have the histology, and indeed I'm against such type of attitude, before doing any endoscopy. It is a pity, because endoscopy in this case is good, because you have a tumor, you have a biopsy, and you have no need to do a liver biopsy. If you have begins, I will say do exploration of the patient by doing a liver biopsy. Liver biopsy is always a small risk. We know that, but it wouldhave been used and useful. Given the fact that anyway you have to do a colonoscopy. And by colonoscopy, you have a biopsy. So we know I think it is important to begin by the endoscopy. I would say exploration before doing a liver biopsy."

Paulo Herman: "What the clinical oncologist does? the first thing they ask is a biopsy."

Rene Adam: "They don't ask for a biopsy of the liver. They ask for a biopsy, if we give them a biopsy of the colon is sufficient."

Rene Adam: [Initial treatment] "We usually begin by four courses—two months—of chemotherapy, and we do another CT scan. In my view, the chemotherapy has not been given according to the recommendation. The recommendation today in front of such type of patient is to do chemotherapy with targeted therapy when what we want is to downsize the lesion. So to say this lesion is resectable. It's a little bit marginally resectable, I think to make a doublet plus a targeted therapy, she's KRAS wildtype, so why not doublet plus cetuximab and panitumumab. Why not? And then we wait or we do a triplet with without targeted therapy. So this patient has only FOLFOX. Sometime it could be sufficient, I agree. But in this case is not sufficient. And so I would prefer rather than to go to upfront surgery to optimize the chemotherapy by adding a targeted therapy."

Hugo Pinto Marques: "What I think is wi this type of lesion that is in the margin of respectability, I think it's very important to start with a very effective regimen. And in fact, I should also agree that perhaps targeted therapy should be should be as first line. The other thing that I my discuss is whether it is useful tomake a biopsy of the lesion at this point, because there is on the of the primary tumor was pretty impressive. And the CEA dropped a lot. So this is to be sure that we are not missing anything else."

Paulo Herman: [treatment after chemotherapy] "I would go for surgery. And I think at that time, if you don't have deprivation available, ALPPS is the only alternative."

Rene Adam: "In view of the possibility of keeping segment IV B, it could change a little bit the things, because I agree that if the volume is not sufficient, probably we should go to an ALPPS. But if we can revise, conserving the segment IVB, it could change a little bit the things because segment IV is hipertrophied due to the right portalembolization."

Hugo Pinto Marques: "I also agree that we should have a chance to preserve part of segment IV B. So I think at this point we all think that probably Liver first approach is the more logical way. At this point you didn't have the possibility of doing a hepatic vein embolization?"

Eduardo Jose Brommelstroet Ramos: "We didn't. It was six years ago."

Hugo Pinto Marques: "So I would probably think this is clearly insufficient. And I wonder, although he has already a portal vein embolization, ALPPS procedure would be useful for this patient."

Olivier Sobrane: "Probably there was several technical options to be discussed. But the main point to me is you didn't reach the oncological target by chemotherapy, you did not obtain any significant reduction in the size, which was probably the most important point to make the surgery simply and less risky for her, but I guess the explanation is because it's mucinous adenocarcinoma so the size did not change."

Eduardo Jose Brommelstroet Ramos: "It was sure."

Rene Adam: "Congratulation for the final good results. Even if I would say, for each decision, an alternative was possible … *all the ways go to Roma*.., you know."
